# Bulimia Nervosa Leading to Squamous Cell Carcinoma of the Esophagus in a Young Adult

**DOI:** 10.7759/cureus.15536

**Published:** 2021-06-08

**Authors:** Shivam Khanna, Dhruv Talwar, Sunil Kumar, Sparsh Madaan, Aditi Goyal

**Affiliations:** 1 Department of Medicine, Jawaharlal Nehru Medical College, Wardha, IND; 2 General Medicine, Acharya Vinobha Bhave Rural Hospital, Wardha, IND; 3 Department of Obstetrics and Gynaecology, Jawaharlal Nehru Medical College, Wardha, IND; 4 Department of Pathology, Jawaharlal Nehru Medical College, Wardha, IND

**Keywords:** bulimia nervosa, squamous cell carcinoma, eating disorders, malignancy, young female

## Abstract

Bulimia nervosa is an eating disorder which is defined as binge eating which is followed by purging and inappropriately increased concern about one’s weight and body shape. We report a case of a 20 year old female who presented with a history of a dysphagia since 2 months. She had a history of binge eating disorder followed by a ritual of purging activity since six years. She also gave a history of increased concern about her weight and body shape. On endoscopy, a growth was seen on the esophagus, which upon biopsy showed features of squamous cell carcinoma.Thus, even in a young female, a simple psychological eating disorder might manifest as a malignancy. This emphasizes the need of early diagnosis and treatment of such innocent looking eating disorders to prevent serious complications in the future.

## Introduction

Bulimia nervosa or bulimia is a psychological disorder of eating which comprises of binge eating activity followed by purging and an increased inappropriate consciousness about one’s weight or shape. Binge eating is characterised by eating large amount of food with increased calories and purging is an activity which comprises of attempt to vomit and remove the consumed calories. There is also increased concern about weight and one’s shape which leads to further anxiety and more purging activities. Other methods to remove the calories consumed by the patient comprises of diuretics, excessive exercise, abuse of diuretics and prolonged fasting. Most individuals with bulimia have weight within normal range [1]. There can be few signs present that point to probable presence of bulimia in the patient, such as hyperpigmentation of the knuckles and loss of enamel of the teeth. This erosion of the teeth is due to gastric acid and hyperpigmentation of knuckles is seen due to forced vomiting. Bulimia might be present as an isolated disorder or along with other disorders like anxiety depression and alcohol abuse. Suicide and self-harm are also reported in patients suffering from bulimia [2]. Squamous cell flat cells lining the esophagus and their carcinoma occurs mostly in the upper and middle half of the oesophagus.

Squamous cell carcinoma is most common esophageal carcinoma around the world. Common risk factors for squamous cell carcinoma include increasing age, achalasia, smoking, alcohol abuse and high starch diet [3]. However, the association of squamous cell carcinoma with eating disorder is rare. We report a case of 20 year old female who presented with the complaint of dysphagia, and on investigations, revealed to be a case of esophageal squamous cell carcinoma linked with bulimia nervosa.

## Case presentation

A 20 year old female presented to the outpatient department with the chief complaint of dysphagia since 2 months which was for solids more than liquids. On detailed history, she revealed binge eating episodes since the last six years which were followed by guilt and episodes of self-provoked vomiting. She also had increased concerns about her body shape and weight. There was no history of any psychiatric illness in the past or any other chronic medical illness. On examination, her pulse was 82 per minute, regular blood pressure was 120/72 mm Hg in right arm supine position, body mass index was 18.2 and respiratory rate was 22 breaths per minute. On systemic examination, chest was bilaterally clear, heart sounds were normal, abdomen was soft and non-tender, and the patient was conscious and oriented. On local examination, there was erosion of dental enamel in the mouth. She was admitted and her lab investigations were within normal range with hemoglobin of 12.3gm/dl, MCV 88, platelet count 1.13 X 10^5^/dl, WBC 6600/dl and normal renal and kidney function tests.

**Figure 1:**
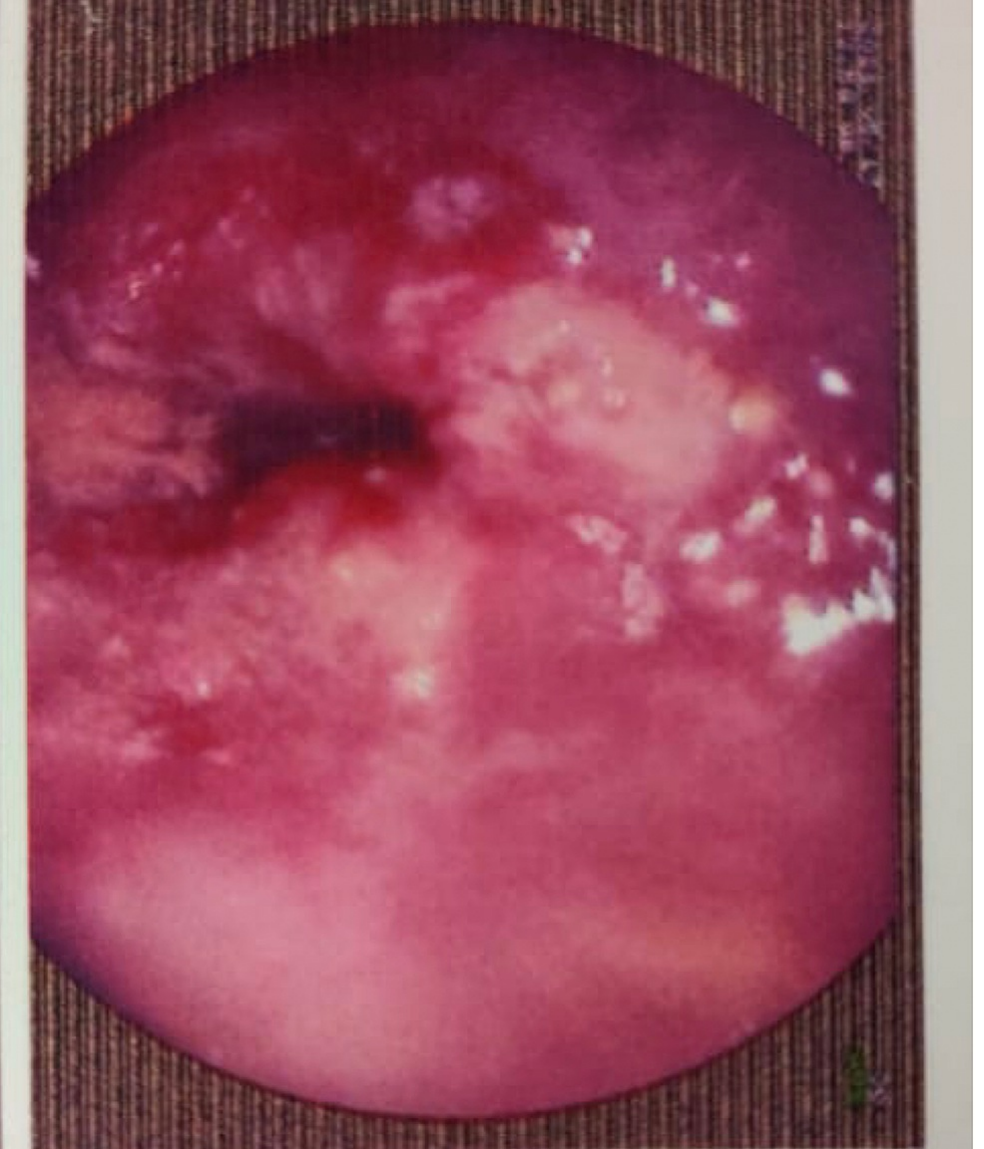
Endoscopy showing Growth in the oesophagus

**Figure 2:**
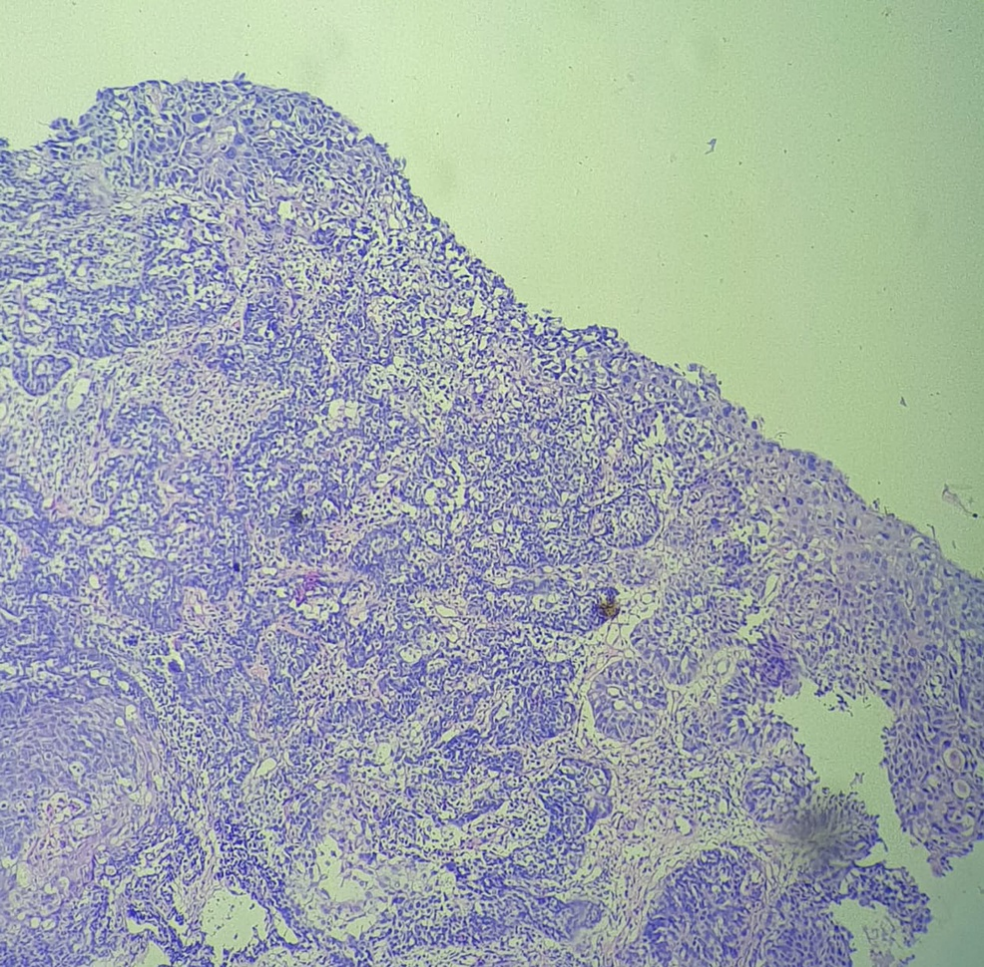
Biopsy Showing Features of Squamous Cell Carcinoma

**Figure 3:**
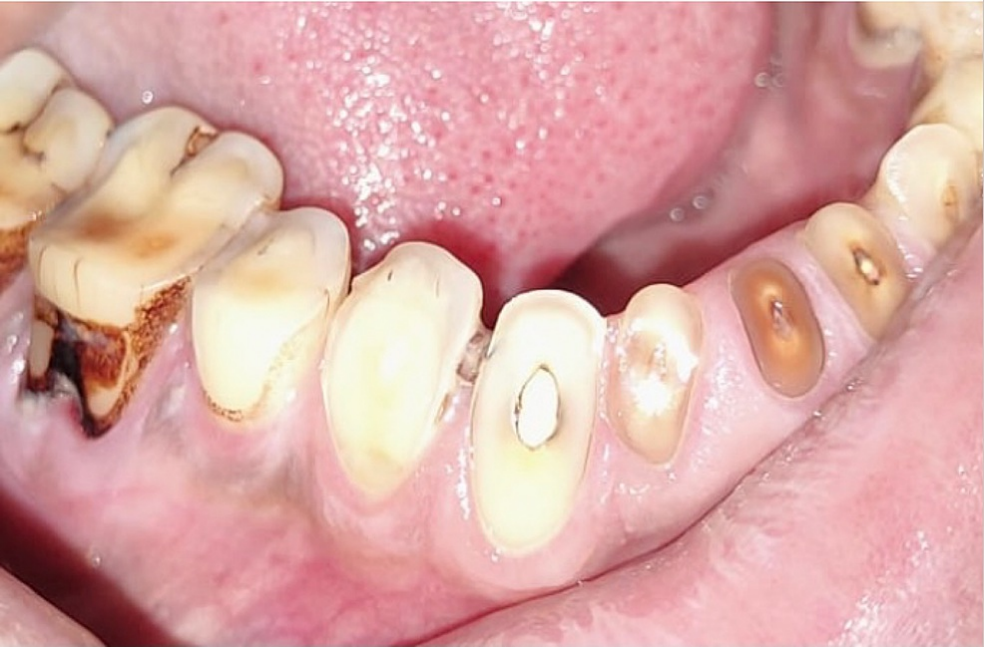
Showing Erosion of enamel due to acid reflux

She was posted for endoscopy which revealed a growth in her esophagus (Figure 1) and a biopsy was taken from the same. Biopsy showed features suggestive of squamous cell carcinoma which was well differentiated (Figure 2). On the basis of investigations and errosion of teeth enamel noted due to reflux acid errosion (figure 3), a diagnosis of long standing bulimia nervosa leading to squamous cell carcinoma of esophagus was established. The patient was initiated on 5-fluorouracil and cisplatin in view of sqamous cell carcinoma along with cognitive behavioural therapy and short serotonin reuptake inhibitors in view of bulimia nervosa and was discharged; she is doing well on follow up.

## Discussion

**Figure 4:**
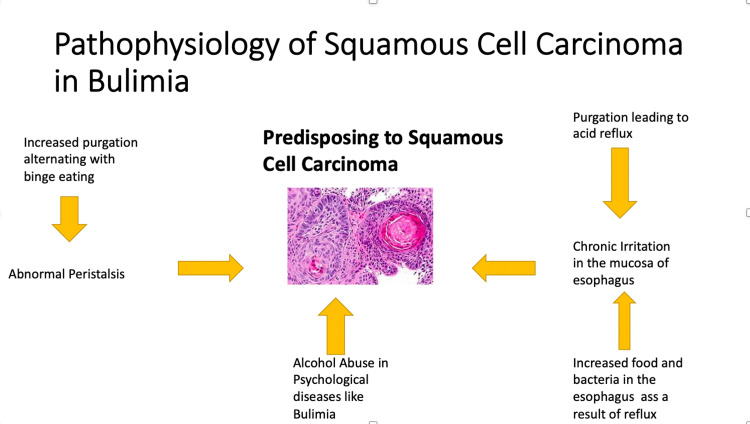
showing pathophysiology of squamous cell carcinoma is bulimia

Squamous cell carcinoma is the most common esophageal cancer which is witnessed throughout the globe. Although more common in the elderly, few cases are reported in the young group as well. With increasing incidence of squamous cell carcinoma, a better knowledge about the associated risk factors can decrease the incidence of this malignancy. While risk factors like achalasia, alcohol and smoking are well established and well known, there is little reporting about eating disorders associated with squamous cell carcinoma. Bulimia nervosa is an eating disorder encountered in young aged females which has increased episodes of binge eating followed by purging and increased concern about one’s external features and weight. With increased purging, there is increased incidence of reflux in the oesophagus [4]. Any factor that leads to increase in oesophageal irritation may lead to an increase in the chances of squamous cell carcinoma. In bulimia nervosa, there is abnormal peristalsis in the oesophagus owing to increased binge eating followed by purging. There are also periods of prolonged fasting involved in bulimia, further complicating the normal peristalsis of oesophagus [5]. This leads to retention of some amount of food or fluids in the oesophagus, promoting bacterial overgrowth. Thus, the regurgitated gastric contents are not cleared. Relation of eating disorders with malignancy is unusual, but an important finding. Cases of anorexia nervosa associated with malignancy have also been reported [7]. However classical cases of bulimia are rare to find and association with squamous cell carcinoma is even more rare [8]. In our case, there was a history of dysphagia to solids which was the presenting complaint of the young female. However, a detailed history allowed the unfolding of the mystery of the etiology behind this presenting complaint which was pointing towards malignancy. It was established that the patient had been suffering from bulimia nervosa for six years which was not diagnosed previously because of her reluctance in contacting a clinician for the same. She had history of typical features of binge eating episodes with increased calorie intake followed by compensatory purging in a practice to remove the excessive calorie intake. This lead to a conclusion of bulimia induced squamous cell carcinoma in our case, which was a rare association in an age group which was unusual. Thus,a proper understanding behind the risk factors for squamous cell carcinoma is needed to control the emerging incidence of this malignancy through the globe and the emerging risk factors for squamous cell carcinoma need to be updated with increasing incidence of psychological eating disorders in today’s age. We postulate that an otherwise neglected eating disorder since six years in a young female led to predisposition to squamous cell carcinoma due to increased irritation of the oesophagus by reflux caused by purging, ultimately leading to a serious symptom of dysphagia which compelled her to present to a clinician (Figure 4).

## Conclusions

We conclude that a neglected eating disorder over the years might lead to a serious complications like squamous cell carcinoma of the esophagus even in a young individual and thus the treating clinicians should be on the lookout for such disorders in young adults to prevent development of serious complications like malignancy in the future.
